# Advancement of cloud-based accounting effectiveness, decision-making quality, and firm performance through digital transformation and digital leadership: Empirical evidence from Vietnam

**DOI:** 10.1016/j.heliyon.2023.e16929

**Published:** 2023-06-02

**Authors:** Bui Quang Hung, Tran Anh Hoa, Tu Thanh Hoai, Nguyen Phong Nguyen

**Affiliations:** a School of Accounting, University of Economics Ho Chi Minh City, Ho Chi Minh City, Viet Nam

**Keywords:** digital transformation, digital leadership, Cloud-based accounting, Decision-making quality, Vietnam

## Abstract

The accounting literature is silent about how digital transformation can be transformed into outstanding business performance through cloud-based accounting effectiveness (CBAE) under the supervision of digital leaders. In the digital age, this mechanism is practically significant for emerging market firms to promote accounting practices and decision-making effectiveness. Thus, this study investigates how CBAE and decision-making quality (DMQ) mediate the impact of digital transformation on firm performance. In addition, the moderating effects of digital leadership on the relationships between digital transformation and CBAE and those between CBAE and DMQ are investigated. The proposed model and its hypotheses are evaluated using partial least squares structural equation modeling (PLS-SEM) on survey data from 252 large-sized Vietnamese firms. The study's findings are as follows: (1) digital transformation positively affects CBAE, which affects DMQ and firm performance; (2) when digital leadership is strong, the effects of digital transformation on CBAE and CBAE on DMQ are amplified. These findings demonstrate how the interaction between digital transformation and digital leadership can contribute to the success of firms in emerging markets that use cloud accounting. In addition, the current study elucidates the mechanism by which digital transformation influences the digitalization of accounting practices and contributes to our understanding of digital transformation research in accounting by introducing digital leadership as a boundary condition.

## 1 Introduction

Accounting and auditing procedures are expensive, time-consuming, and arduous [1]. As information technology has evolved, manual accounting systems are no longer sufficient to meet decision-making information requirements [2]. Therefore, utilizing new accounting information systems and tools has radically altered the accounting profession's efficiency [3]. However, with new technologies, accountants can now devote more time to complex analyses and achieve statistical accounting with a greater capacity to monitor and assess the financial health of a firm [4]. As cloud computing is one of the technologies that will continue to dominate the agenda of the accounting profession for many years [5], cloud-based accounting (CBA) can significantly disrupt the accounting sector [6].

In the context of globalization, technological advancements, and the proliferation of Internet platforms [7], the creation of the CBA is deemed essential. CBA is an Internet-based virtue accounting information system. It provides online accounting services along with accounting administration and accounting decision-making [8]. CBA was specifically developed to manage financial information [9], which is expected to contribute to successful firms with various competitive benefits. CBA offers valuable features, such as simple access and better data-processing capabilities, than traditional systems [10]. In addition to enhancing data-sharing and reducing unnecessary rekeying and associated data-entry errors, CBA provides authorized access with an Internet connection and a web browser with remote access [11]. The development of CBA is the creation of a new platform for accounting software that does not require installation on a local computer [12]; therefore, CBA reduces expenses by eliminating upfront software and hardware costs [13]. If information from CBA is effectively utilized in decision-making, it can significantly and positively impact firm performance [14]. Further, CBA could reduce the burden associated with business continuity and disaster recovery plans, making it an attractive option for risk management [11] and improving performance [15]. Given its competitive benefits, it is reasonable to assume that a growing number of firms will move to CBA. This trend is even more apparent in the context of an increasing need for remote work due to COVID-19 [16].

In line with fast-growing CBA adoption, practitioners have conducted a more robust body of research on CBA than academics [17], with some evidence from the accounting literature regarding several antecedents of CBA adoption. For instance, Ma et al. [6] have indicated that drivers of CBA adoption include the perceived benefits of programs of partners (e.g. information technology [IT] vendors), organizational readiness (e.g. financial resources, IT sophistication, and trading partner readiness), and pressures from external parties (e.g. trading partners and cloud providers). In addition, Altin and Yilmaz [18] have found that users’ attitudes toward using CBA also affect the adoption of CBA. Although this body of research has enhanced our understanding of methods to promote CBA adoption, there is still a shortage of knowledge regarding a mechanism for integrating digital transformation and digital leadership to increase CBAE. The current study fills this gap in our understanding by exploring how digital transformation affects CBAE toward improving decision-making quality (DMQ) and firm performance. In addition, this study examines the moderating role of digital leadership in the chain of relationships between digital transformation, CBAE, DMQ, and firm performance.

Digital transformation refers to a fundamental change process facilitated by the innovative application of digital technology and the strategic exploitation of essential resources and competencies to enhance a firm significantly and redefine its value offer for its stakeholders [19]. Leadership aspects are essential in this process [19]. In recent years, as digital technologies have rapidly transformed organizations and industries, the concept of digital leadership has been introduced to address the competencies that leaders must develop in the current digital age [20]. Digital leadership is the process of achieving strategic digitalization success for a firm and within its business ecosystem [21]. Digital leadership is distinct from conventional leadership and is marked by a more agile and flexible leadership style, a strong aptitude for new technologies, digital literacy, an openness to innovation, and a digital culture lived by leaders [22].

Previous studies have investigated the nexus between digital transformation and digital leadership (e.g. Refs. [[23], [24], [25], [26]]). For example, Abbu et al. [23] have asserted that digital leadership might be a powerful facilitator of digital transformation processes because digital leaders can instill organizational confidence in these innovative and occasionally risky digital transformation endeavors. Magesa and Jonathan [26] have found that digital leadership is expected to drive digital transformation successfully by supporting economic growth, promoting innovation and entrepreneurship, and enhancing service delivery. In addition, a recent study by Fernandez-Vidal et al. [25] has found that digital leadership skills, as one type of human capital, play a critical role in digital transformation by driving business change, mastering fluid and loose organizational structure, and developing talent complexity in the context of the digital era. However, our review shows a limited understanding of how digital leadership and digital transformation can interact to promote CBAE and DMQ––the ability of a firm to make accurate and appropriate decisions to firm performance [27]. This gap is both practical and significant because bridging it can provide insights into a mechanism for firms to foster accounting practices and decision-making effectiveness under digital leadership in the digital transformation context.

Drawing on upper echelon theory (UET) [28], this study bridges the above gaps by developing and testing a moderated mediation model consisting of (1) the serial mediating effects of digital transformation on firm performance via CBAE and DMQ and (2) the moderating effect of digital leadership on the digital transformation––CBAE and CBAE––DMQ links. UET asserts that executives’ backgrounds, traits, and experiences significantly impact organizational decision-making and performance outcomes [28]. This paper argues that digital leadership is among the upper-echelon characteristics that influence organizational strategic choices that support cloud accounting information systems. In other words, it is possible to accelerate the ways digital transformation can be converted to enhance firm performance via CBAE under the strong directions of digital leaders. Our study makes significant contributions to the limited literature regarding the role of top management in digital transformation efforts with the unexplored “view from the top” (e.g. Ref. [25]).

The remainder of the paper is organized as follows. The next section discusses the theoretical background, emphasizing the chain of effects between digital transformation, CBAE, DMQ, firm performance, and the moderating effect of digital leadership on this chain. Following that, the research methodology and results are presented, and the theoretical and managerial implications of the study are then discussed. Finally, the conclusion section presents the limitations of this study and proposes directions for future research.

## 2 Theoretical background, model, and hypothesis development

### 2.1 Upper echelon theory

In accordance with the UET, which links organizational strategic choices and performance to managerial characteristics, the adoption of new technology necessitates adapting business processes that could not be performed without the participation of upper management [29]. According to the UET, leaders' characteristics (e.g. cognitive biases and personal values) influence decision-makers’ strategic decisions [30]. For example, information technology skills (or the lack thereof) are considered a cognitive bias that can influence leaders’ innovation-related strategic decisions [31]. Thus, UET is relevant in explaining how digital leaders can moderate the process through which digital transformation affects CBAE, DMQ, and firm performance. Specifically, in our research, the UET can help explain how digital leadership strengthens the relationships between digital transformation on CBAE and between CBAE and DMQ.

### 2.2 Digital transformation and CBAE

CBA results from the digital transformation of accounting from traditional to more autonomous accounting information systems [32]. Digital transformation aims to improve a firm by triggering significant changes to its properties through combinations of information, computing, communication, and connectivity technologies [33]. Furthermore, digital transformation helps firms improve their ability to collect, disseminate, store, analyze, and display data to enhance optimal data-processing ability [34].

The above premise posits that digital transformation can promote the effectiveness of CBA. Specifically, some digital transformation measures (e.g. the degree to which data is digitalized, the extent to which different processes are linked using digital technologies, the efficiency of the customer interface with digitality, and the level of information exchange internally with digitality) [35] can make accounting tasks in the cloud simpler, more specialized, and easier to document, and accounting information is better communicated across department functions. These consequences are definite measures of CBA effectiveness [15]. In light of the preceding discussion, the following hypothesis is presented:

H1. Digital transformation has a positive effect on CBAE.

### 2.3 CBAE and DMQ

Compared to a locally managed, traditional accounting system, CBA offers greater scalability in response to customer demand, requires minimal capital expenditure, and enables superior cost management [36]. In addition, accounting in the cloud can provide firms with numerous advantages over more conventional information systems, including improved real-time data-processing capabilities and ease of access [10,13]. An effective CBA can integrate all the critical information required for accounting processes into a single system [37]. Another study by Altin and Yilmaz [18] has found that CBA applications can facilitate rapid, secure communication and generate standard reports.

In addition, the advanced collaboration features of CBA, such as online communication tools, shared workspaces, and real-time data access, are expected to increase agility and prompt business decision-making [38]. Thus, cloud accounting offers advantages such as enhanced decision-making [1]. These advantages of CBA can result in a higher-quality information system, which is conducive to a higher DMQ [14]. In addition, correct, accurate, and reliable information can be a reflective indicator of a high-quality accounting system in the cloud [14]. This system can ensure high-quality accounting information while decreasing the effort of the decision-making process [39]. Based on the preceding arguments, this study proposes the following hypothesis:

H2. CBAE has a positive effect on DMQ.

### 2.4 DMQ and firm performance

DMQ relates to the correctness and precision of decisions [27]. It refers to how well actual results from a decision meet internal expectations [40]. Quinn et al. [41] have found that high-level DMQ based on cloud accounting can promote cost-effectiveness for firms. Consequently, the effectiveness of such decision-making can positively influence firm performance and be a driver of firm value [14]. In addition, information derived from a cloud-based accounting system can be of high quality. This high-quality information can inform better decisions and enhance firm performance [42]. Accordingly, this study proposes the following hypothesis:

H3. DMQ has a positive effect on firm performance.

Based on the above arguments and the well-established relationship between digital transformation and firm performance in the context that firms are increasingly transforming themselves to become more agile by integrating and exploiting the benefits of digital transformation [43], this study suggests the following hypothesis:

H4. CBAE and DMQ serially mediate the effect of digital transformation on firm performance.

### 2.5 The moderating role of digital leadership

In the technology landscape, digital leaders have become critical for firms to optimize resources and performance [44,45]. Digital leadership can help a firm succeed in digital strategy and the business ecosystem [21]. Notably, digital leadership embraces new ideas that boost digital progress in areas such as employment, market access, product commercialization, and knowledge acquisition and by strategically deploying the firm's information technology assets to enhance its business outcomes [46]. In addition, digital leadership is related to implementing and using management strategies compatible with the digital age, including a reliance on contemporary technology platforms [45,46] and activities related to CBA. Thus, digital leadership is critical to equipping a firm with the necessary conditions to enhance CBAE through digital transformation. In doing so, digital leadership does what is best for the success of the firm's technology strategy by leveraging digital transformation and divergent thinking about technology in management [21]. According to UET, digital leadership could impact the distribution of firm attention towards digital technologies [47,48] to maintain and develop CBA. As a result, in firms with more substantial digital leadership levels, the effect of digital transformation on CBAE can be strengthened. Based on the previous arguments driven by UET, this study hypothesizes the following.

H5. Digital leadership positively moderates the effect of digital transformation on CBAE.

The technology era is characterized by the comprehensive application of digital technologies to organizational systems, decision-making, and participants; CBA is no exception. In this context, under the moderating role of digital leadership, DMQ can be improved through CBA [14]. Indeed, the CBA can integrate all the important information [37] and generate standard reports [18]. Firms with CBAE will aggregate and transform information faster in decision-making, thereby improving DMQ. However, digital leaders will further enhance this outcome. This is because, under the guidance of digital leaders, a firm can easily leverage CBAE to enhance DMQ [1,14] and other activities to accelerate growth [26].

On the other hand, digital leadership can boost leaders' commitment to transparency, explainability, and data-informed decision-making [23]; therefore, this study argues that, for firms with a high level of digital leadership, the impact of CBAE on the quality of decision-making can be enhanced. In addition, the UET posits that strong leaders are the primary factor in influencing and implementing an organization's strategic decisions [28]. Therefore, it can be argued that leaders with strong digital competencies can successfully leverage the digitalization of accounting practices to support decision-making by promoting their echelon characteristics. In this context, CBAE can inform strategic decision-making more effectively. More precisely, digital leadership will enhance the positive impact of CBAE on DMQ. Based on the preceding arguments, the following hypothesis is proposed:

H6. Digital leadership positively moderates the effect of CBAE on DMQ.

**Fig. 1:**
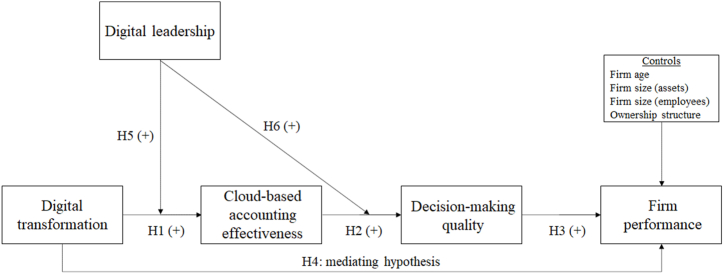
Proposed model and hypotheses.

Fig. 1 shows the proposed model and the corresponding hypotheses.

## 3 Methods

### 3.1 Research methods

#### 3.1.1 Research site

Vietnam was selected as the research site for our study because of the potential growth of digitalization and cloud computing in this emerging country. Vietnam's market for cloud computing is anticipated to expand by approximately 26% per year, the fastest rate in Southeast Asia and significantly higher than the global average of 16% [49]. Approximately 40 firms now offer cloud-based computing services in the Vietnamese market. These include multinationals such as Google, Microsoft, and Amazon; significant, large firms such as Viettel, VNPT, CMC, and FPT; and several smaller, application-focused companies [49]. In addition, Vietnam's national digital transformation strategy has also outlined the fundamental objective that, by 2025, the digital economy will account for 20% of gross domestic product (GDP) and, by 2030, it will account for approximately 30% of GDP [50]. According to this strategy, Vietnam will become a digital nation by 2030.

#### 3.1.2 Participants

Because the unit of analysis is at the firm level, only one informant represented each firm to answer the survey questionnaires. The target informants had to satisfy the following filter criteria: (1) work in large-sized firms that use cloud services (e.g. FPT, Viettel-CHT, CMC, IBM, Microsoft, Mat Bao, SAP) for accounting practices, (2) be top-level or mid-level managers, (3) have at least two years of experience in their firms, and (4) have work experience in digitalization and CBA. The email list of the potential informants for the survey was compiled using personal emails extracted from the professional social network LinkedIn, which has been utilized to obtain the emails of potential respondents in previous studies (e.g. Ref. [51]).

#### 3.1.3 Instruments

Well-established scales from the literature were used to measure the main variables in our study. Specifically, digital transformation was assessed using a five-item scale adapted from Nasiri et al. [35]. Next, the 13-item scale Magesa and Jonathan [26] adopted was used to measure digital leadership. This scale has four dimensions: inspirational role (five items), innovation role (three items), absorbing uncertainty role (three items), and visionary role (two items). CBAE was measured using an eight-item scale adapted from Cleary and Quinn [15]. Informants were also asked to evaluate their firms' DMQ based on the outcome of their decisions, following the four-item scale adapted from Al-Okaily et al. [14]. As collecting objective performance data using financial reports is rather difficult, this study used a subjective performance scale adapted from Liang and Gao [52] to measure firm performance. Subsequently, informants were required to compare their firms’ performance with that of their main competitors over the previous three years in terms of market share, new customer acquisition, customer satisfaction, sales, return on investment, and overall profitability. All of these scale items have seven anchors, except those for CBAE, which had five. Finally, following Zhu et al. [53] and Tu and Nguyen [54], this study employed firm size in terms of assets and full-time equivalent employees, firm age, and ownership structure (1 = without foreign capital; 2 = with foreign capital) as control variables of firm performance. The measurement scales of the main constructs are shown in Table 2.

#### 3.1.4 Procedure

Sale items included in the survey form were back-translated into Vietnamese using the method suggested by Brislin [55]. The interval between the two phases was three months. This short time interval was chosen to minimize memory bias and the drop rate in our survey [56]. Data were collected in two phases to minimize common method bias with the single-informant approach [57]. The online questionnaire was sent to the emails of the potential informants using SurveyMonkey. All participants had been provided informed consent for this study, as they could respond voluntarily to the survey questionnaire and leave at any time if they felt uncomfortable. A unique identification code was assigned to each respondent to facilitate matching responses.

In phase 1, an email was sent to 2356 informants, who were asked to complete filter questions, provide demographic information, and respond to questions regarding digital transformation, digital leadership, and CBAE. After phase 1, 367 complete responses were obtained. In stage 2, conducted three months later, phase 1's informants were asked to provide information regarding DMQ and firm performance; 252 valid responses were obtained, with a final response rate of 10.70% [252/2356 × 100%]. This rate is acceptable for email survey research in Vietnam. Following the recommendation of Armstrong and Overton [58], in both phases, this study conducted independent *t*-tests for potential nonresponse bias, which revealed no differences between the first and fourth quartiles of responses regarding demographic and main variables. This result indicates a low risk of nonresponse bias in our study.

Table 1 summarizes the demographic information of informants and participating firms. Forty-eight percent of the sampled firms were in the services sector, followed by the trading sector (27.4%) and the manufacturing sector (24.6%); this industry structure in the sample accurately reflects the economic structure of Vietnam, in which the service sector contributes more than 51% of GDP [59]. The relatively low participation of companies without foreign capital (32.1%) compared to those with foreign capital (67.9%) in the sample appropriately reflects the fact that Vietnamese firms account for less than 20% of the market share for cloud computing [60].

**Table 1:** Demographic information (*n* = 252).

	Frequent	Percent		Frequent	Percent
*Position*			*Full-time equivalent employees*		
Top-level managers	86	34.1	201–500	129	51.2
Mid-level managers	166	65.9	501–1000	40	15.9
*Tenure (years)*			1001–5000	60	23.8
2–5	124	49.2	5001–10,000	12	4.8
6–10	71	28.2	>10,000	11	4.4
11–20	46	18.3	*Total assets (VND billion)*		
>20	11	4.4	101–200	82	32.5
*Industry type*			201–500	74	29.4
Manufacturing	62	24.6	501–1000	62	24.6
Trading	69	27.4	>1000	34	13.5
Services	121	48.0	*Firm age (years)*		
*Ownership structure*			≤5	37	14.7
Without foreign capital	81	32.1	6–10	48	19.0
With foreign capital	171	67.9	11–20	75	29.8
			21–50	73	29.0
			>50	19	7.5

## 4 Results

### 4.1 Scale evaluation

To evaluate the reliability of the scale items, their outer loadings and *t*-values were computed. Table 2 shows that most outer loadings (ranging between 0.69 and 0.93) were above the cut-off value of 0.70, and their corresponding *t*-values were higher than 1.96 (ranging between 17.00 and 104.90). The only item from the CBAE scale (“It has simplified our accounting processes”) has a loading of 0.69, slightly less than the cut-off value; it was nonetheless retained to ensure the content validity of this scale. Moreover, all the variables have a composite reliability (CR) (ranging between 0.82 and 0.95) above the recommended value of 0.70 and an average variance extracted (AVE) ranging between 0.52 and 0.80 of more than the cut-off value of 0.50 [61]. These results indicate that all the scales had a satisfactory level of reliability.

**Table 2:** Scale items and evaluation.

	Outer loading	*t*-value
Digital transformation [35] (CR = 0.95; AVE = 0.80)		
We aim to digitalize everything that can be digitized	0.93	93.63
We collect massive volumes of data from different sources	0.93	81.22
We aim to create stronger networking between the different business processes with digital technologies	0.93	104.90
We aim to enhance an efficient customer interface with digitality	0.87	37.89
We aim to achieve information exchange with digitality	0.80	21.28
Digital leadership [26]		
*Inspirational role (CR = 0.89; AVE = 0.61)*		
Unusually able to persuade others of their viewpoint	0.74	17.19
Capacity to influence the organization and convince others to influence	0.76	18.56
Demonstrates and imparts strong positive emotions for work	0.81	38.94
Deserves trust and can be believed and relied upon to keep their word	0.81	38.24
Stimulates others to put forth efforts above and beyond the call of duty and make personal sacrifices	0.76	24.42
*Innovation role (CR = 0.87; AVE = 0.68)*		
Anticipates, attempts to forecast events, considers what will happen in the future	0.84	31.78
Prepared to meet emerging business challenges, anticipates and responds to new paradigms of competition, navigates complexity and leverages data and analytics to make decisions	0.80	23.79
Willing to invest major resources in endeavors that do not have a high probability of success	0.84	24.37
*Absorbing uncertainty role (CR = 0.88; AVE = 0.72)*		
Acts on good judgment and practical ideas or understanding	0.86	35.99
Communicates with others frequently	0.82	31.24
Provides vision and purpose	0.86	31.99
*Visionary role (CR = 0.87; AVE = 0.77)*		
Has a vision and imagination of the future	0.88	64.96
Gives courage, confidence, or hope through reassuring and advising	0.87	44.90
CBAE [15] (CR = 0.90; AVE = 0.52)		
It has limited our ability to customize accounting and finance systems to our needs (R)	0.72	24.94
It has made our daily accounting tasks more standardized	0.74	23.59
It has simplified our accounting processes	0.69	20.45
It has made it easier for any accounting staff member to perform any accounting task	0.75	26.50
It has made it easier to communicate accounting procedures to new accounting and finance staff	0.70	23.06
It has made it easier to document accounting procedures	0.72	25.61
It has made it easier to adapt accounting procedures	0.72	21.93
It has made it easier to replicate accounting and finance systems to other parts or branches of the organization	0.75	25.99
DMQ [14] (CR = 0.82; AVE = 0.53)		
*Based on the information from CBA, the outcome of the decisions that my company makes is usually*		
correct (the outcome may have minor errors)	0.71	19.05
accurate (the outcome has no errors at all)	0.71	17.00
precise (CBA will lead to the same outcome every time the company faces the same problem)	0.74	21.80
Dependable	0.75	25.83
Firm performance [52] (CR = 0.93; AVE = 0.69)		
Customer satisfaction	0.85	61.79
Market share	0.84	49.81
New customer acquisition	0.84	50.30
Return on investment	0.81	41.23
Sales revenue	0.82	46.09
Overall profitability	0.82	46.68

Notes: CR: composite reliability; AVE: Average variance extracted; R: Reversed code.

Table 3 shows the discriminant validity analysis of the main variables. Following Fornell and Larcker [62], this study compared the squared root of the AVE of these variables with the correlations between them. For each variable, its squared root of the AVE was higher than all its correlations with other variables, suggesting good discriminant validity of the main variables. Along with Fornell and Larcker's procedure [62], the more stringent Heterotrait–Montrait (HTMT) test [63] was used, with HTMT values ranging from 0.06 to 0.87. Since these values are significantly less than 0.90, discriminant validity is reaffirmed [63].

**Table 3:** Discriminant validity analysis.

	1___	2___	3___	4___	5___	6___	7___	8___
1. Digital transformation	**0.90**							
2. Inspirational role	(0.12)	**0.78**						
	*0.13*							
3. Innovation role	(0.01)	0.24**	**0.83**					
	*0.06*	*0.30*						
4. Absorbing uncertainty role	(0.14)*	0.26**	0.30**	**0.85**				
	*0.16*	*0.31*	*0.39*					
5. Visionary role	(0.15) *	0.72**	0.18**	0.23**	**0.88**			
	*0.18*	*0.87*	*0.24*	*0.31*				
6. Cloud-based accounting	0.47**	0.36**	0.26**	0.23**	0.31**	**0.72**		
	*0.52*	*0.42*	*0.31*	*0.27*	*0.39*			
7. Decision-making quality	0.41**	0.24**	0.18**	0.16*	0.25**	0.65**	**0.73**	
	*0.51*	*0.31*	*0.24*	*0.21*	*0.35*	*0.82*		
8. Firm performance	0.13*	0.10	0.05	0.03	0.12	0.25**	0.37**	**0.83**
	*0.15*	*0.11*	*0.09*	*0.06*	*0.14*	*0.28*	*0.46*	

Notes: First value = correlation between variables (off-diagonal); second value (italic) = HTMT ratio; square root of average variance extracted (bold diagonal); *, **: correlations are significant at the 5% and 1% levels, respectively (two-tailed *t*-test).

### 4.2 Common method bias and multicollinearity issues

Because this study used a single-informant approach to collect data, it was possible that common method bias could distort the relationships among the variables [57]. Thus, this study employed the marker-variable technique [64] using a single item, “I am satisfied with my life in general,” which was intentionally included in the questionnaire. The results indicate that, when the effects of the shared correlation due to common method variance (CMV; *rM*) were partialled, the mean change in the correlations of the main constructs, i.e. the gap between the uncorrected correlation (*rU*) and the CMV-adjusted correlation (*rA*), was insignificant at 0.02. Therefore, our study has a low risk of common method bias. This study then analyzed the variance inflation factor (VIF) values of the independent variables [65] to evaluate the possibility of multicollinearity. As the inner VIF values ranged from 1.02 to 1.93, well below the criterion of ten, no significant multicollinearity issues were identified.

### 4.3 Hypothesis-testing results

In this study, four hierarchical models were run in partial least square structural equation modeling (PLS-SEM) to test the proposed model and hypothesis. Model 1 shows the relationship between digital transformation and firm performance, and Model 2 adds CBAE as the mediator of this relationship. Model 3 shows the augmentation of Model 2 with the addition of DMQ in the relationship between CBAE and firm performance. Model 4 was the final and complete model; it was the same as Model 3 but included digital leadership as the moderator. Table 4 shows the indices to evaluate the paths between variables (i.e. β correlation coefficient and *t*-value) and the adjusted R^2^ values of the dependent variables, which were computed using 5000 bootstrapping times in PLS-SEM. The adjusted R^2^ values ranged between 0.17 and 0.53, higher than the recommended threshold of 0.10 to justify the acceptance of the variance of a dependent variable [66].

**Table 4:** Hypothesis-testing results.

		Model 1	Model 2 (with CBAE as the mediating variable)		Model 3 (with CBAE and DMQ as the mediating variables)			Model 4 (with CBAE and DMQ as the mediating variables and DL as the moderating variable)		
Dependent variable		FP	CBAE	FP	CBAE	DMQ	FP	CBAE	DMQ	FP
	Independent variable									
H1	DT	0.15 (2.67)^c^	0.47 (8.02)	c0.03 (0.45)	0.47 (7.81)^c^	0.14 (2.55)^b^	−0.02 (0.31)	0.52 (10.66)^c^	0.12 (2.09)^b^	−0.02 (0.32)
	DL							0.44 (7.36)^c^	0.19 (3.06)^c^	
H2	CBAE			0.23 (3.77)^c^		0.58 (13.16)^c^	0.03 (0.37)		0.53 (9.00)^c^	0.03 (0.39)
H3	DMQ						0.35 (4.76)^c^			0.34 (4.62)^c^
	DL × DT								0.30 (5.11)^c^	
	DL × CBAE									0.19 (4.24)^c^
	Control variable									
	Assets	0.15 (2.17)^b^		0.17 (2.59)^c^			0.20 (3.02)^c^			0.20 (2.78)^c^
	Employees	0.08 (0.93)		0.06 (0.82)			0.03 (0.49)			0.03 (0.44)
	Firm age	0.26 (4.84)^c^		0.25 (4.99)^c^			0.24 (5.17)^c^			0.24 (5.23)^c^
	Ownership	0.04 (0.58)		0.03 (0.49)			0.05 (0.80)			0.05 (0.81)
Adjusted R2		0.17	0.22	0.20	0.22	0.43	0.26	0.53	0.47	0.26
Indirect effect								Estimate	LLCI	ULCI
H4	DT→CBAE→DMQ							0.28 (6.72)^c^	0.20	0.36
	CBAE→DMQ→FP							0.18 (3.99)^c^	0.10	0.28
	DT→CBAE→DMQ→FP							0.08 (3.60)^c^	0.05	0.15

*Notes*: DT: digital transformation; DL: digital leadership; CBA: cloud-based accounting; DMQ: decision-making quality; FP: firm performance; DL × DT: interaction between DL and DT; DL × CBAE: interaction between DL and CBAE; numbers in brackets: *t*-values; ^a, b^, and ^c^ denote significance at 10%, 5%, and 1%, respectively (two-tailed *t*-test).

H1 conjectured that digital transformation positively influences CBAE, and this hypothesis was supported (model 1: β = 0.47; *t*-value = 8.02). Our analysis also provided support for H2, proposing that CBAE has a positive effect on DMQ (model 2: β = 0.58; *t*-value = 13.16). H3 proposed that DMQ positively affects firm performance, and this hypothesis was supported (model 3: β = 0.35; *t*-value = 4.76).

To test H4 regarding the serial mediating effects of CBAE and DMQ on the relationship between digital transformation and firm performance, three specific indirect effects (i.e. of the mediator) of the paths from digital transformation to firm performance were calculated (see Table 4). These effects were significant (β ranging between 0.08 and 0.28; t-value ranging between 3.60 and 6.72), and their confidence intervals did not contain zero. Moreover, when the moderating variables (i.e. CBAE and DMQ) were added to the relationship between digital transformation and firm performance, the effect of digital transformation and firm performance became insignificant (model 2: β = 0.03; *t*-value = 0.45; model 3: β = −0.02; *t*-value = 0.31). Therefore, the mediating effects of CBAE and DMQ were found, and H4 was supported.

**Fig. 2:**
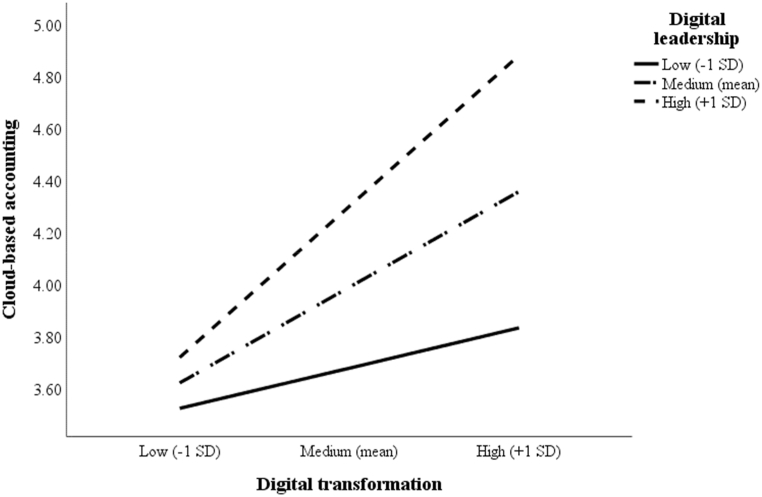
Interaction effect of digital transformation and digital leadership on CBAE.

**Fig. 3:**
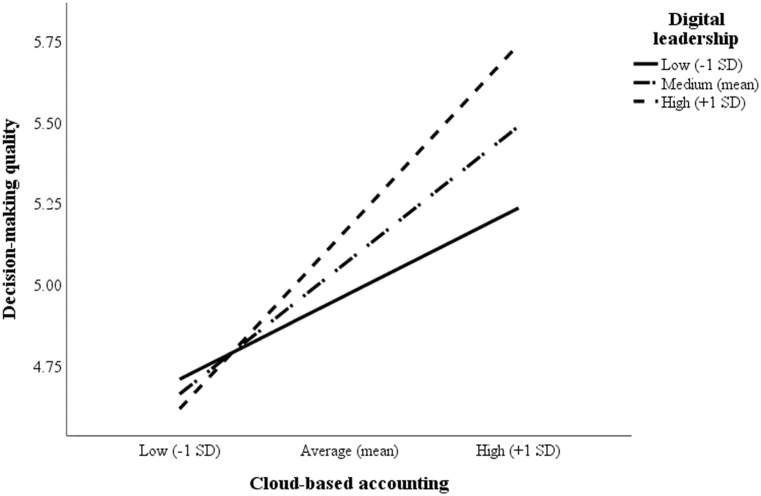
Interaction effect of CBAE and digital leadership on DMQ.

To test H5 and H6 regarding the moderating effects of digital leadership on the two relationships (1) between digital transformation and CBAE and (2) between CBAE and DMQ, two interaction terms were created: DL × DT and DL × CBAE (see Table 4). These terms were formed by multiplying the interacting variables after mean-centering them, thereby avoiding multicollinearity issues [67]. The effects of DL × DT on CBAE (model 4: β = 0.30; *t*-value = 5.11) and of DL × CBAE on DMQ (model 4: β = 0.19; *t*-value = 4.24) were both significant, confirming H5 and H6. To further illustrate the significance of the interactions, the effects of digital transformation on CBAE and of CBAE on DMQ were plotted for low (˗1 SD), medium (mean), and high (+1 SD) levels of digital leadership (see Fig. 2, Fig. 3). According to Fig. 2, Fig. 3, these effects are more profound for firms with a higher degree of digital leadership than for those with medium or lower levels of social innovation.

## 5 Implications and future research directions

This study examines the moderating effects of digital leadership on how digital transformation impacts firm performance via CBAE and DMQ. Using the UET, our hypotheses were developed and tested using data from a questionnaire survey of large-sized Vietnamese firms. The results indicate that digital transformation positively impacts CBAE, increasing DMQ and firm performance. The findings also indicate that the influence of digital transformation on CBAE and that of CBAE on DMQ are stronger with higher levels of digital leadership. Our study has some theoretical and managerial implications, which are discussed in the following sections.

### 5.1 Theoretical and managerial implications

This study has two theoretical implications. First, the findings add to the limited research in accounting information systems on the mechanism to convert digital transformation into improved firm performance. Specifically, the mediating roles of CBAE and DMQ in the relationship between digital transformation and firm performance help clarify this mechanism. This is an interesting addition to the literature on cloud accounting in the context of the digital age (e.g. Refs. [5,6,15,41]). Second, our study demonstrates that the moderating effect of digital leadership on the serial mediating impacts of digital transformation on CBAE, DMQ, and firm performance cannot be ignored, thereby contributing to extant accounting research grounded in the UET [28]. Third, our study advances our understanding of the conditions under which digital transformation is beneficial in influencing CBA practices and decision-making utilizing CBA system data. In particular, this study demonstrates that digital leadership favorably moderates the digital transformation–CBAE–DMQ chain. Thus, to our knowledge, our study is the first to empirically evaluate the boundary conditions of the influence of digital leadership. This enhances the scientific understanding of the significance of digital leadership in developing countries’ environments.

Our research also has two managerial implications. First, the results show that digital transformation enhances CBAE, which in turn affects DMQ, and that these serial impacts are greatly amplified by higher degrees of digital leadership, which is critical for managers in developing nations such as Vietnam. These findings are relevant because large Vietnamese firms can use the interplay between digital transformation and digital leadership to advance CBAE, improve decision-making processes, and enhance firm performance. Second, our findings clarify the repercussions of digital transformation, which can help firms drive the success of their cloud-based accounting practices. Overall, the significance of the research issue on how digital transformation and digital leadership can be combined to promote accounting in the cloud suggests that this study can broaden our theoretical understanding and provide managerial guidance for firms in other emerging economies.

### 5.2 Limitations and future research directions

Our research has several limitations. First, although two phases were employed to collect data, a single-source approach may not guarantee that our results are free from common method bias [57]. Second, this study could not make causal claims because data were collected with a time lag of only three months between the dependent and independent variables and because this study did not manipulate variables or use randomly assigned methods. Future studies could overcome this restriction by adopting a longitudinal or experimental design with a longer time lag between collecting data on the dependent and independent variables. Second, this study employed subjective measures to assess firm performance. Accordingly, it is possible that managers were biased in their reporting of firm performance. Consequently, it would be intriguing if future studies evaluated firm success using more objective sources (e.g. financial statements). In addition, Vietnamese companies were sampled without regard to industry categorization. Considering that different industrial sectors may have varying degrees of digitization and CBA adoption, it would be more appropriate for future research to analyze industry classifications as control variables. As our study was done in Vietnam, the findings should be interpreted in the context of a developing economy. Although Vietnam has many features of an emerging economy, other emerging countries may have distinct and diverse contextual variables (e.g. politics, culture, government investment in technology) that could provide greater insight and inform the development of theories. Therefore, it would be beneficial for future studies to validate the suggested model using data from many countries to explore the potential effects of unique elements of the local setting.

## 6 Conclusion

The current study examined how digital transformation impacts CBAE, DMQ, and firm performance utilizing survey data from 252 large Vietnamese firms. In addition, the confirmation of our proposed model demonstrated that digital leadership influences this process by moderating the digital transformation–CBAE–DMQ chain. These results contribute to the body of knowledge in the following ways: First, the paper contributes to the literature on digital transformation by highlighting its role in promoting accounting efficiency and decision-making to improve firm performance. Second, the findings contribute to the literature on leadership by defining digital leadership as the boundary condition of the digital transformation–CBAE–DMQ chain. In terms of practical contributions, firms in emerging markets should focus on the cultivation of digital transformation to promote accounting effectiveness and decision-making for performance improvement. This includes the digitalization of business processes, the collection of massive volumes of data from various sources, the adoption of digital technologies in connecting different business processes, and the improvement of customer interface and information exchange through digitality. Moreover, emerging market firms must promote all roles of digital leadership (i.e. inspirational, innovation, absorbing uncertainty role, and visionary), which are important moderating factors that enhance the effectiveness of digital transformation in fostering accounting decision-making and firm performance.

## Author contribution statement

Bui Quang Hung: Conceived and designed the experiments; Performed the experiments; Analyzed and interpreted the data; Contributed reagents, materials, analysis tools or data; Wrote the paper. Tu Thanh Hoai: Conceived and designed the experiments; Performed the experiments; Analyzed and interpreted the data; Contributed reagents, materials, analysis tools or data; Wrote the paper. Tran Anh Hoa: Conceived and designed the experiments; Performed the experiments; Analyzed and interpreted the data; Contributed reagents, materials, analysis tools or data; Wrote the paper. Nguyen Phong Nguyen: Conceived and designed the experiments; Performed the experiments; Analyzed and interpreted the data; Contributed reagents, materials, analysis tools or data; Wrote the paper.

## Data availability statement

Data will be made available on request.

## Declaration of interest's statement

The authors declare no competing interests.

## Declaration of competing interest

The authors declare that they have no known competing financial interests or personal relationships that could have appeared to influence the work reported in this paper.

## References

[1] Cai C.W. (2021). Triple‐entry accounting with blockchain: how far have we come?. Account. Finance.

[2] Al-Hattami H.M., Hashed A.A., Kabra J.D. (2021). Effect of AIS success on performance measures of SMEs: evidence from Yemen. Int. J. Bus. Inf. Syst..

[3] Schmitz J., Leoni G. (2019). Accounting and auditing at the time of blockchain technology: a research agenda. Aust. Account. Rev..

[4] Zhang Y., Xiong F., Xie Y., Fan X., Gu H. (2020). The impact of artificial intelligence and blockchain on the accounting profession. IEEE Access.

[5] Yigitbasioglu O., Green P., Cheung M.-Y.D. (2023). Digital transformation and accountants as advisors. Accounting, Auditing and Accountability Journal.

[6] Ma D., Fisher R., Nesbit T. (2021). Cloud-based client accounting and small and medium accounting practices: adoption and impact. Int. J. Account. Inf. Syst..

[7] Dimitriu O., Matei M. (2014). A new paradigm for accounting through cloud computing. Procedia Econ. Finance.

[8] Zhang Y., Su X., Xie L., Zhong Y., Chan D. (2015). Environmental Science and Information Application Technology.

[9] Yau-Yeung D., Yigitbasioglu O., Green P. (2020). Cloud accounting risks and mitigation strategies: evidence from Australia. Account. Forum.

[10] Oliveira T., Thomas M., Espadanal M. (2014). Assessing the determinants of cloud computing adoption: an analysis of the manufacturing and services sectors. Inf. Manag..

[11] Dimitriu O., Matei M. (2015). Cloud accounting: a new business model in a challenging context. Procedia Econ. Finance.

[12] Sultan N. (2014). Making use of cloud computing for healthcare provision: opportunities and challenges. Int. J. Inf. Manag..

[13] Asatiani A., Apte U., Penttinen E., Rönkkö M., Saarinen T. (2019). Impact of accounting process characteristics on accounting outsourcing-Comparison of users and non-users of cloud-based accounting information systems. Int. J. Account. Inf. Syst..

[14] Al-Okaily M., Alghazzawi R., Alkhwaldi A.F., Al-Okaily A. (2022). The effect of digital accounting systems on the decision-making quality in the banking industry sector: a mediated-moderated model. Glob. Knowl. Mem. Commun..

[15] Cleary P., Quinn M. (2016). Intellectual capital and business performance: an exploratory study of the impact of cloud-based accounting and finance infrastructure. J. Intellect. Cap..

[16] CPA (2021). https://www.cpaaustralia.com.au/-/media/project/cpa/corporate/documents/tools-and-resources/financial-reporting/business-technology-report-2021.pdf?rev=b99ab28c4c9b4e299402b9fd010a8d8f.

[17] Atanasovski A., Toceva T. (2022). Research trends in disruptive technologies for accounting of the future - a bibliometric analysis. Accounting & Management Information Systems.

[18] Altin M., Yilmaz R. (2021). Adoption of cloud-based accounting practices in Turkey: an empirical study. Int. J. Publ. Adm..

[19] Gong C., Ribiere V. (2021). Developing a unified definition of digital transformation. Technovation.

[20] Schiuma G., Schettini E., Santarsiero F., Carlucci D. (2021). The transformative leadership compass: six competencies for digital transformation entrepreneurship. Int. J. Entrepreneurial Behav. Res..

[21] El Sawy O.A., Kræmmergaard P., Amsinck H., Vinther A.L. (2016). How LEGO built the foundations and enterprise capabilities for digital leadership. MIS Q. Exec..

[22] Wirtz B.W. (2021).

[23] Abbu H., Mugge P., Gudergan G. (2022). Successful digital leadership requires building trust. Res. Technol. Manag..

[24] Abbu H., Mugge P., Gudergan G., Hoeborn G., Kwiatkowski A. (2022). Measuring the human dimensions of digital leadership for successful digital transformation: digital leaders can use the authors' digital leadership scale to assess their own readiness and ability to accelerate digital transformation. Res. Technol. Manag..

[25] Fernandez-Vidal J., Perotti F.A., Gonzale R., Gasco J. (2022). Managing digital transformation: the view from the top. J. Bus. Res..

[26] Magesa M.M., Jonathan J. (2022). Conceptualizing digital leadership characteristics for successful digital transformation: the case of Tanzania. Inf. Technol. Dev..

[27] Ghasemaghaei M. (2019). Does data analytics use improve firm decision making quality? The role of knowledge sharing and data analytics competency. Decis. Support Syst..

[28] Hambrick D.C., Mason P.A. (1984). Upper echelons: the organization as a reflection of its top managers. Acad. Manag. Rev..

[29] Caluwe L., De Haes S. (2019). Board level it governance: a scoping review to set the research agenda. Inf. Syst. Manag..

[30] Manner M.H. (2010). The impact of CEO characteristics on corporate social performance. J. Bus. Ethics.

[31] Héroux S., Fortin A. (2018). The moderating role of IT-business alignment in the relationship between IT governance, IT competence, and innovation. Inf. Syst. Manag..

[32] Özcan E.Ç., Akkaya B. (2020). Agile Business Leadership Methods for Industry 4.0.

[33] Vial G. (2019). Understanding digital transformation: a review and a research agenda. J. Strat. Inf. Syst..

[34] Verhoef P.C., Broekhuizen T., Bart Y., Bhattacharya A., Dong J.Q., Fabian N., Haenlein M. (2021). Digital transformation: a multidisciplinary reflection and research agenda. J. Bus. Res..

[35] Nasiri M., Ukko J., Saunila M., Rantala T. (2020). Managing the digital supply chain: the role of smart technologies. Technovation.

[36] Marston S., Li Z., Bandyopadhyay S., Zhang J., Ghalsasi A. (2011). Cloud computing—the business perspective. Decis. Support Syst..

[37] Penttinen E., Halme M., Lyytinen K., Myllynen N. (2018). What influences choice of business-to-business connectivity platforms?. Int. J. Electron. Commer..

[38] Romero D., Vernadat F. (2016). Enterprise information systems state of the art: past, present and future trends. Comput. Ind..

[39] Gonzales R., Wareham J., Serida J. (2015). Measuring the impact of data warehouse and business intelligence on enterprise performance in Peru: a developing country. J. Global Inf. Technol. Manag..

[40] Visinescu L.L., Jones M.C., Sidorova A. (2017). Improving decision quality: the role of business intelligence. J. Comput. Inf. Syst..

[41] Quinn M., Strauss E., Kristandl G. (2014). The effects of cloud technology on management accounting and business decision-making. Financ. Manag..

[42] DeLone W.H., McLean E.R. (2016). Information systems success measurement. Foundations Trends® in Information Systems.

[43] Nguyen N.P., Tu T.H. (2022). The impacts of digital transformation on data-based ethical decision-making and environmental performance in Vietnamese manufacturing firms: the moderating role of organizational mindfulness. Cogent Bus. Manag..

[44] Ahlquist J. (2014). Trending now: digital leadership education using social media and the social change model. J. Leader. Stud..

[45] Brett J. (2019). Evolving Digital Leadership: How to Be a Digital Leader in Tomorrow's Disruptive World.

[46] AlAjmi M.K. (2022). The impact of digital leadership on teachers' technology integration during the COVID-19 pandemic in Kuwait. Int. J. Educ. Res..

[47] Saputra N., Hutajulu G.E. (2020). Engaging the millennials at office: tracking the antecedents of holistic work engagement. Pol. J. Manag. Stud..

[48] Wang X., Li Y., Tian L., Hou Y. (2023). Government digital initiatives and firm digital innovation: evidence from China. Technovation.

[49] Vietnamplus, 2020, IT, telecoms development trends identified. Retrieved from https://en.vietnamplus.vn/it-telecoms-development-trends-identified/233512.vnp.

[50] Minister (2020). https://english.luatvietnam.vn/decision-no-749-qd-ttg-on-approving-the-national-digital-transformation-program-until-2025-with-a-vision-184241-doc1.html.

[51] Mintz O., Currim I.S. (2013). What drives managerial use of marketing and financial metrics and does metric use affect performance of marketing-mix activities?. J. Market..

[52] Liang X., Gao Y. (2020). Marketing performance measurement systems and firm performance. Eur. J. Market..

[53] Zhu Y., Sun L.-Y., Leung A.S. (2014). Corporate social responsibility, firm reputation, and firm performance: the role of ethical leadership. Asia Pac. J. Manag..

[54] Tu T.H., Nguyen N.P. (2022). Internal control systems and performance of emerging market firms: the moderating roles of leadership consistency and quality. Sage Open.

[55] Brislin R.W. (1970). Back-translation for cross-cultural research. J. Cross Cult. Psychol..

[56] Einarsen S., Hoel H., Notelaers G. (2009). Measuring exposure to bullying and harassment at work: validity, factor structure and psychometric properties of the Negative Acts Questionnaire-Revised. Work. Stress.

[57] Podsakoff P.M., MacKenzie S.B., Lee J.-Y., Podsakoff N.P. (2003). Common method biases in behavioral research: a critical review of the literature and recommended remedies. J. Appl. Psychol..

[58] Armstrong J.S., Overton T.S. (1977). Estimating nonresponse bias in mail surveys. J. Market. Res..

[59] CIA.gov (2021). https://www.cia.gov/the-world-factbook/countries/vietnam/.

[60] Vietnamplus (2020). https://en.vietnamplus.vn/vietnam-enterprises-hold-20-percent-of-domestic-cloud-market-share/231416.vnp.

[61] Nunnally J.C., Bernstein I.R. (1994).

[62] Fornell C., Larcker D.F. (1981). Structural equation models with unobservable variables and measurement error: algebra and statistics. J. Market. Res..

[63] Henseler J., Ringle C.M., Sarstedt M. (2015). A new criterion for assessing discriminant validity in variance-based structural equation modeling. J. Acad. Market. Sci..

[64] Lindell M.K., Whitney D.J. (2001). Accounting for common method variance in cross-sectional research designs. J. Appl. Psychol..

[65] O'Brien R.M. (2007). A caution regarding rules of thumb for variance inflation factors. Qual. Quantity.

[66] Falk R.F., Miller N.B. (1992).

[67] Aiken L.S., West S.G., Reno R.R. (1991).

